# Submucosal Buccal Nodule: A Rare Case of Oral Dirofilariasis

**DOI:** 10.1590/0037-8682-0101-2026

**Published:** 2026-09-25

**Authors:** Low Yin Kuan, Mohd Farifzul Anuar Mohd Zuki, Tan Chuey Chuan, Wanninayake M Tilakaratne, Nurul Izyan Zainuddin

**Affiliations:** 1 Universiti Malaya, Faculty of Dentistry, Department of Oral and Maxillofacial Clinical Sciences, Kuala Lumpur, Malaysia.

A 46-year-old Indonesian woman presented with a persistent swelling on her right cheek for one week. Clinical examination revealed a localized, well-defined firm, and movable subcutaneous nodule measuring approximately 2x2 cm (Figure 1 **)**. The nodule was also palpable intraorally through bimanual palpation. The dental panoramic radiograph (Figure 2 **)** showed no signs of mandibular bone resorption or abnormal radiopaque or radiolucent lesions in the right mandible.

**FIGURE 1:**
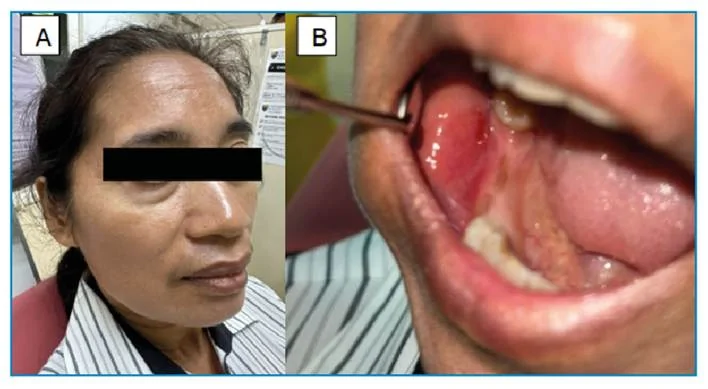
Clinical presentation of the lesion. **(A)** A subtle subcutaneous nodule observed over the right cheek. **(B)** The corresponding submucosal nodule was palpable on bimanual palpation, with mild focal erythema of the overlying right buccal mucosa.

**FIGURE 2:**
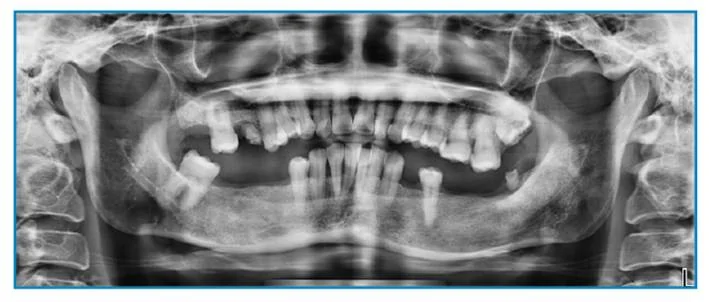
Dental panoramic radiograph showing a partially edentulous lower jaw with retained roots of teeth 16 and 38. No abnormal radiopaque or radiolucent lesions are observed.

An excisional biopsy was performed, and histopathological examination revealed a soft tissue nodule with a dense infiltrate of chronic inflammatory cells, predominantly eosinophils, lymphocytes, and histiocytes. A coiled nematode, characterized by thick external cuticles with longitudinal ridges resembling a cogwheel, was identified within the fibrous connective tissue (Figure 3 **)**. The absence of uterine tubes indicated that the parasite was an adult male. A diagnosis of dirofilariasis was made, and the patient recovered without complications post-surgery. 

**FIGURE 3:**
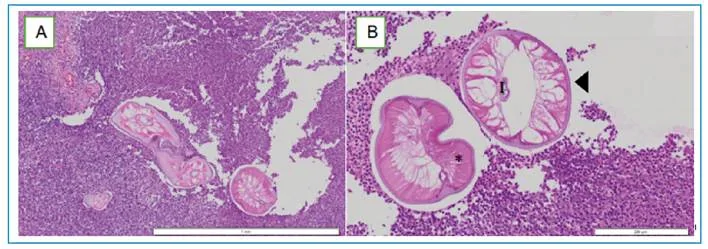
Histopathological features of the lesion. **(A)** Parasitic worms scattered within a dense inflammatory infiltrate rich in eosinophils (hematoxylin and eosin, ×40). **(B)** Transverse section of the nematode at ×100 magnification showing thick musculature (asterisk), a corrugated cuticle (arrowhead), and the intestine (I).

Human dirofilariasis is a mosquito-borne zoonosis in which humans serve as dead-end hosts^1^. Oral involvement is rare and commonly presents as a solitary submucosal nodule resembling benign soft tissue lesions^2^. This rare case highlights the need to consider parasitic infections in the differential diagnosis of oral soft tissue swellings, particularly in patients from endemic areas or those in close contact with dogs. Early recognition and histopathological confirmation are crucial for accurate diagnosis and to avoid unnecessary extensive surgical procedures. Surgical excision is both diagnostic and curative.

## References

[1] Joseph M, Krishna MM, Vijayan A (2023). Human subcutaneous dirofilariasis. Cureus.

[2] Jayasinghe R, Tilakaratne WM, Tilakaratne WM, Kallarakkal TG (2023). Clinicopathological correlation of oral diseases.

